# The aging lung: tissue telomere shortening in health and disease

**DOI:** 10.1186/s12931-018-0794-z

**Published:** 2018-05-11

**Authors:** Stephanie Everaerts, Elise J. Lammertyn, Dries S. Martens, Laurens J. De Sadeleer, Karen Maes, Aernoud A. van Batenburg, Roel Goldschmeding, Coline H. M. van Moorsel, Lieven J. Dupont, Wim A. Wuyts, Robin Vos, Ghislaine Gayan-Ramirez, Naftali Kaminski, James C. Hogg, Wim Janssens, Geert M. Verleden, Tim S. Nawrot, Stijn E. Verleden, John E. McDonough, Bart M. Vanaudenaerde

**Affiliations:** 1 Laboratory of Respiratory Diseases, Department of Chronic Diseases, Metabolism & Aging (CHROMETA), KU Leuven, Herestraat 49, O&NI, box 706, B-3000 Leuven, Belgium; 2 Centre for Environmental Sciences, Hasselt University, Hasselt, Belgium; 3 Department of Pulmonology, St Antonius ILD Center of Excellence, St Antonius Hospital, Nieuwegein, the Netherlands; 4 Department of Pathology, University Medical Center Utrecht, Utrecht, the Netherlands; 5 Division of Heart and Lungs, University Medical Center Utrecht, Utrecht, the Netherlands; 6 Department of Respiratory Diseases, University Hospitals Leuven, Leuven, Belgium; 7 Section of Pulmonary, Critical Care, and Sleep Medicine, Yale University, New Haven, CT USA; 8 University of British Columbia James Hogg Research Centre, St. Paul’s Hospital, Vancouver, BC Canada; 9 Department of Public Health & Primary Care, KU Leuven, Leuven, Belgium

**Keywords:** Cystic fibrosis, Chronic obstructive pulmonary disease, Chronic hypersensitivity pneumonitis, Chronic lung allograft dysfunction, BOS, RAS, Cellular senescence, Telomere length

## Abstract

**Background:**

Telomere shortening has been associated with several lung diseases. However, telomere length is generally measured in peripheral blood leucocytes rather than in lung tissue, where disease occurs. Consequently, telomere dynamics have not been established for the normal human lung nor for diseased lung tissue. We hypothesized an age- and disease-dependent shortening of lung tissue telomeres.

**Methods:**

At time of (re-)transplantation or autopsy, 70 explant lungs were collected: from unused donors (normal, *n* = 13) and patients with cystic fibrosis (CF, *n* = 12), chronic obstructive pulmonary disease (COPD, *n* = 11), chronic hypersensitivity pneumonitis (cHP, *n* = 9), bronchiolitis obliterans syndrome (BOS) after prior transplantation (n = 11) and restrictive allograft syndrome (RAS) after prior transplantation (*n* = 14). Lungs were inflated, frozen and then scanned using CT. Four tissue cores from distinct lung regions were sampled for analysis. Disease severity was evaluated using CT and micro CT imaging. DNA was extracted from the samples and average relative telomere length (RTL) was determined using real-time qPCR.

**Results:**

The normal lungs showed a decrease in RTL with age (*p* < 0.0001). Of the diseased lungs, only BOS and RAS showed significant RTL decrease with increasing lung age (*p* = 0.0220 and *p* = 0.0272 respectively). Furthermore, we found that RTL showed considerable variability between samples within both normal and diseased lungs. cHP, BOS and RAS lungs had significant shorter RTL in comparison with normal lungs, after adjustment for lung age, sex and BMI (*p* < 0.0001, *p* = 0.0051 and *p* = 0.0301 respectively). When investigating the relation between RTL and regional disease severity in CF, cHP and RAS, no association was found.

**Conclusion:**

These results show a progressive decline in telomere length with age in normal, BOS and RAS lungs. cHP, BOS and RAS lungs demonstrated shorter RTL compared to normal lungs. Lung tissue RTL does not associate with regional disease severity within the lung. Therefore, tissue RTL does not seem to fully reflect peripheral blood telomere length.

**Electronic supplementary material:**

The online version of this article (10.1186/s12931-018-0794-z) contains supplementary material, which is available to authorized users.

## Background

Aging is a complex biological process characterized by progressive decline of all physiological functions resulting in a time-dependent increase in mortality [1, 2]. With increasing age, the respiratory system undergoes structural remodelling, including rearrangement of extracellular matrix, dilatation of alveoli and enlargement of airspaces leading to lung function decline. This process of structural aging together with immunosenescence leads to an increased susceptibility to acute and chronic pulmonary disease, which can be influenced by individual factors, such as genetic background and exposure [3, 4].

One of the defining mechanisms behind aging is related to telomere shortening [5, 6]. Telomeres are stretches of repetitive DNA capping the ends of chromosomes, protecting them from unscheduled DNA degradation. In somatic cells, telomeres undergo attrition at each cell replication until the point they become dysfunctional, inducing a DNA damage response leading to cellular senescence. Shortened peripheral blood leukocyte telomeres have been associated with various age-related diseases, such as atherosclerosis [7] and type 2 diabetes mellitus [8], and an increased risk for some cancers [9]. This suggests that the increased turnover and replication of circulating leukocytes during chronic inflammation accelerates the rate of leukocyte telomere shortening [7, 10].

In patients with respiratory disorders such as chronic obstructive pulmonary disease (COPD) and interstitial lung disease (ILD), shorter peripheral blood leukocyte telomeres have been demonstrated compared to healthy individuals [11–13]. Moreover, mutations in essential telomerase genes, telomerase reverse transcriptase (TERT) and RNA template (TR), are associated with idiopathic pulmonary fibrosis (IPF) and COPD [14, 15]. Furthermore, a single-nucleotide polymorphism of MUC5B, which is associated with both IPF and chronic hypersensitivity pneumonitis (cHP), associates with shorter blood leucocyte telomere length in cHP [16]. However, much less is known about telomere length within the lung tissue, where cell turnover is low compared to blood leucocytes [17]. Moreover, telomere dynamics in healthy aging lung have not been established.

We hypothesized an age- and disease-dependent shortening of lung tissue telomeres, as has been shown in blood leucocyte telomeres. Explanted lung tissue was used, based on the availability in our lung transplant center. First, relative telomere length (RTL) in unused donor lungs was measured to determine telomere length in normal human lung tissue. Second, RTL was measured in lung tissue of patients suffering from end-stage chronic lung diseases including COPD, cHP, cystic fibrosis (CF) and two phenotypes of chronic lung allograft dysfunction (CLAD) after prior lung transplantation. Finally, we investigated the association between regional disease severity within the lung and RTL.

## Methods

### Study material

Based on the availability in our lung transplant center, an assortment of 70 explant lungs was collected between 2009 and 2015, including 13 unused donor lungs, which were used to determine telomere length in normal human lung tissue. These lungs were obtained after decline for lung transplantation (LTx) by the handling surgeon due to persistent (micro) thrombi despite flushing (*n* = 4), unexpected death of the recipient (*n* = 1), mild contusion (*n* = 2), presence of a kidney tumour in the donor (n = 2), suspicion of emphysema (n = 1), beginning fibrosis (n = 1), infection (n = 1) or rupture of an artery (n = 1). Two donor lungs of contrasting age, which were declined because of edema and infection, were collected for an additional experiment. According to Belgian law, organs from prospective donors, which are of insufficient quality for LTx and have been conclusively declined by the transplant surgeon, can be used for research purposes. CF (*n* = 12), COPD (*n* = 11) and cHP (*n* = 9) explant lungs were collected at the time of LTx. CLAD lungs (*n* = 25), subdivided into bronchiolitis obliterans syndrome (BOS) (n = 11) and restrictive allograft syndrome (RAS) (*n* = 14), were collected during re-LTx or autopsy. Diagnostic criteria are listed in Additional file 1. All LTx patients gave written informed consent to use their lungs for research purposes. The study was approved by the Medical Ethics Board of University Hospitals Leuven, Belgium (ML6385).

### Lung tissue processing

All lungs were processed as previously described [18, 19]. In brief, the main stem bronchus was cannulated and lungs were inflated to near total lung capacity, maintained at 10 cm water pressure and subsequently frozen in liquid nitrogen vapours before storing at − 80 °C. High resolution computed tomography (HRCT) scan was taken of all frozen lungs. Lungs were cut into 2 cm-thick slices from apex to base with cores (diameter 1.4 cm) systematically removed using a drill press. In this study, four cores per lung were analysed: two cores from apical slices (slice number 2 to 5) and two cores from basal slices (slice number 6 to 12), randomly selected to reflect the spatial heterogeneity within the lung. In CF, an additional distinction was made between cores taken from an area with structural abnormalities including bronchiectasis, airway destruction and increased tissue density on HRCT, and normal-appearing tissue. Tissue of unused donor lungs was processed in the same way, except that areas with suspicion of any abnormality were strictly avoided while sampling.

### Micro CT analysis

Micro CT scanning was used to obtain high-resolution images of lung tissue and measure disease severity in the cores. Frozen cores were scanned using a Bruker Skyscan 1172 micro CT device (Bruker, Kontich, Belgium) with a resolution of 10 μm while maintained at − 30 °C, using a cooling stage. Scans were reconstructed using NRecon and images analysed using CTAn software (Bruker). Normal lung parenchyma with a well-developed alveolar structure has a high surface area to volume ratio (surface density). Consequently, measurement of surface density was used to determine the extent of normal tissue within each sample with decreasing values reflecting loss of normal tissue through disease (e.g. emphysema in COPD or fibrosis in cHP/RAS). Cores of RAS and cHP lungs were stratified based on the median surface density value of their disease group.

### Relative telomere length measurement

DNA was extracted from a portion of each lung tissue core (height: 0.5 cm, diameter: 0.7 cm) using the QIAamp DNA Mini Kit (Qiagen Inc., Venlo, The Netherlands). DNA concentration and purity were determined using NanoDrop (Thermo Scientific NanoDrop Technologies, Wilmington, Delaware, USA) and DNA integrity was checked using agarose gel electrophoresis. Average RTL was measured using a modified quantitative real-time PCR (qPCR) protocol as described previously [20]. Details are provided in Additional file 1.

### Fluorenscent in situ hybridization

An extensive description of the processing for fluorescent in situ hybridization (FISH) is provided in Additional file 1. Telomere labelling was performed on tissue slices using a telomere-Cy3 PNA Probe (Panagene, Daejeon, South-Korea). Alveolar type 2 (AT2) cells were labelled with pro-SPC staining (AB3786,1/500,Merck Millipore, Darmstadt, Germany), DNA of the tissue slides was stained using 4′,6-diamidino- 2-phenylindole (DAPI, 25 μg/mL).

A Fluorescence microscope (Leica DM 5500B) at high magnification (63×) was used for image capture. Multiple images per slice were taken within 24 h after staining, all pro-SPC positive cells were used. Mean relative telomere signal per cell was calculated as the total Cy3 area divided by the DAPI signal per cell.

### Statistical analyses

All statistical analyses were performed using SAS 9.4 (SAS Institute Inc., Cary, NC, USA) and R 3.2.2 statistical software (R Foundation for Statistical Computing, Vienna, Austria). Graphical representation of data was generated using GraphPad Prism 4.0 Software (GraphPad Software, San Diego, CA, USA) and R 3.2.2. Prior to analyses, RTL of each core was log_10_ transformed to normalize the dataset. Demographic and clinical characteristics were compared by Kruskal-Wallis analysis with Dunn’s correction for multiple testing in case of continuous variables and Chi^2^ for discrete variables. The association of tissue core RTL with lung age was analysed by mixed linear models with the original lung as random effect and adjustment for BMI and sex. Paired t-test analysis was used to investigate regional differences within the groups. RTL comparison between specific diseases and normal lungs was performed with a mixed linear model with the original lung as random effect, adjusted for lung age, sex and BMI. An unpaired t-test was performed to compare tissue cores with normal versus abnormal appearance (in CF) and mild versus severe disease (in cHP and RAS). A mixed model with the original lung as random effect was used to assess the association between core RTL and surface density, while accounting for lung age, BMI and sex. The coefficient of variation (CV) was calculated per disease group (inter-lung variability) and within the lungs (intra-lung variability). *P*-values < 0.05 were considered significant in all analyses.

## Results

### Patient characteristics

Demographic and clinical characteristics are summarized in Table 1. Lung age reflects the calendar age of the transplanted subject or the lung donor in case of CLAD. Lung function of the normal lungs was unknown. CF lung age was significantly lower than age of normal lungs. Patients with CF, BOS or RAS had a significantly lower BMI compared to donors of unused normal lungs. Other diseases showed no significant differences compared to characteristics of normal lungs.

**Table 1:** Demographic and clinical characteristics of lungs

	NORMAL	CF	COPD	cHP	BOS	RAS
Subjects, n	13	12	11	9	11	14
Lung age, years	48 (20)	23 (7)*	60 (3)	58 (10)	27 (24)	46 (23)
Lung age, range	16-72	19-33	48-61	36-61	16-48	9-61
Male, n (%)	10 (77)	5 (42)	5 (45)	4 (44)	5 (45)	8 (57)
Male donor, n (%)	NA	NA	NA	NA	5 (45)	8 (57)
Patient height, cm	175 (15)	162 (18)	163 (22)	166 (19)	168 (12)	168 (17)
Patient weight, kg	80 (25)	46 (14)***	60 (17)	74 (9)	51 (21)**	60 (21)*
BMI, kg/m^2^	25 (4)	18 (2)***	21 (8)	27 (4)	18 (5)**	19 (6)**
FEV_1_, L	NA	0.8 (0.4)	0.8 (0.5)	1.1 (0.8)	0.6 (0.3)	0.9 (0.5)
FEV_1_, % predicted	NA	23 (10)	31 (11)	49 (21)	20 (3)	26 (12)
FVC, L	NA	1.6 (0.8)	2.0 (0.4)	1.3 (0.9)	1.5 (1)	1.4 (0.6)
FVC, % predicted	NA	45 (15)	66 (33)	38 (20)	46 (20)	33 (13)
FEV_1_/FVC	NA	0.5 (0.1)	0.4 (0.1)	0.9 (0.2)	0.4 (0.1)	0.7 (0.3)
DLco, % predicted	NA	38 (33)^a^	33 (14)	30 (7)	40 (18)^b^	35 (8)^c^

^a, b, c^ respectively 6, 1 and 7 missing values. Results are given as n (%) or median (IQR). % predicted of FEV1 and FVC was based on ECSC equations before 2012 [45] and on GLI equations from 2012 onwards [46]. ATS recommendations were used for equations of DLCO reference [47]. Significant difference with the normal lungs is indicated with *p < 0.05, **p < 0.01 and ***p < 0.001

*CF* cystic fibrosis, *COPD* chronic obstructive pulmonary disease, *cHP* chronic hypersensitivity pneumonitis, *BOS* bronchiolitis obliterans syndrome, *RAS* restrictive allograft syndrome, *NA* not applicable, *BMI* body mass index, *FEV1* forced expiratory volume in 1 s, *FVC* forced vital capacity, *FEV1/FVC* tiffeneau index, *DLco* diffusion capacity of the lung for carbon monoxide

### Relative telomere length in normal lung tissue

**Fig. 1:**
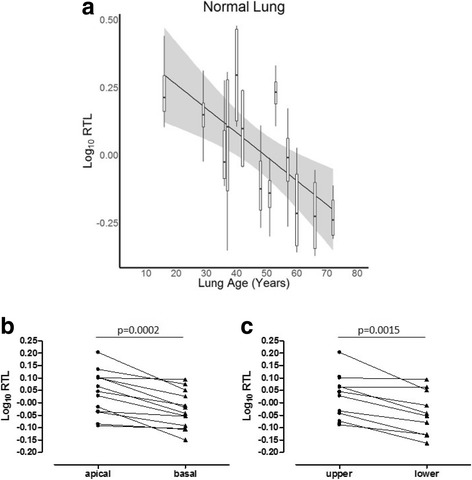
RTL decrease with age and regional difference in normal lung tissue. **a**: log_10_ RTL versus lung age in normal lungs. Log_10_ RTL per lung is presented as a boxplot with the grey area representing the 95% confidence interval. **b** and **c**: Paired t-test of log_10_ RTL in normal lungs based on spatial distribution. Every dot shows the mean log_10_ RTL of cores originating from the respective region per lung. **b**: difference in log_10_ RTL between cores originating from the apical (*n* = 26) and basal (n = 26) lung regions (*p* = 0.0002). **c**: difference in log_10_ RTL between upper (*n* = 37) and lower lobe (*n* = 15) cores (*p* = 0.0015)

Unused donors were between 16 and 72 years of age. Each 1-year increase in age was associated with a significant decrease in RTL (*p* < 0.0001) (Table 2). Figure 1a and Table 3 show the inter- and intra-lung variance in RTL for normal lungs (CV of 18.3 and 16.3% respectively). RTL of cores originating from apical lung slices was longer compared to basal cores (*p* = 0.0002) (Fig. 1b). Also, when considering lobar distribution, upper lobe tissue had longer RTL compared to lower lobe tissue (*p* = 0.0015) (Fig. 1c).

**Table 2:** Relation between RTL and lung age in normal and diseased lung tissue

Group	Estimate	% change	95% CI	*p*-value
NORMAL	−0.0039	−0.90	−1.26 - -0.54	**0.0001**
CF	−0.0045	−1.04	−2.21 - 0.16	0.090
COPD	0.015	3.41	1.36 - 5.49	**0.0016**
cHP	−0.0024	− 0.55	−4.98 - 4.10	0.81
BOS	−0.0033	−0.76	−1.41 - -1.41	**0.022**
RAS	−0.0021	−0.47	− 0.88 - -0.06	**0.027**

Mixed linear models were used to measure the association between tissue RTL and lung age, with the lung as random effect, adjusted for sex and BMI. Estimates are presented as percentage change (95% CI) in average RTL for each 1-year increase in lung age

*RTL* relative telomere length, *95% CI* 95% confidence interval, *CF* cystic fibrosis, *COPD* chronic obstructive pulmonary disease, *cHP* chronic hypersensitivity pneumonitis, *BOS* bronchiolitis obliterans syndrome, *RAS* restrictive allograft syndrome

*p*-value < 0.05 is captured in bold

**Table 3:** Coefficients of variation (%) for RTL between and within lungs

	NORMAL	CF	COPD	cHP	BOS	RAS	Mean
CV between lungs for each group, %	18.3	12.5	17.6	30	22.4	18.0	19.8
Mean CV within lung, %	16.3	11.2	17.3	15.2	11.9	12.4	14.1

Results are presented as percentages. Coefficients of variation were calculated per disease group (based on mean lung RTL values) and per lung (based on core RTL values)

*RTL* relative telomere length, *CV* coefficient of variation, *CF* cystic fibrosis, *COPD* chronic obstructive pulmonary disease, *cHP* chronic hypersensitivity pneumonitis, *BOS* bronchiolitis obliterans syndrome, *RAS* restrictive allograft syndrome

**Fig. 2:**
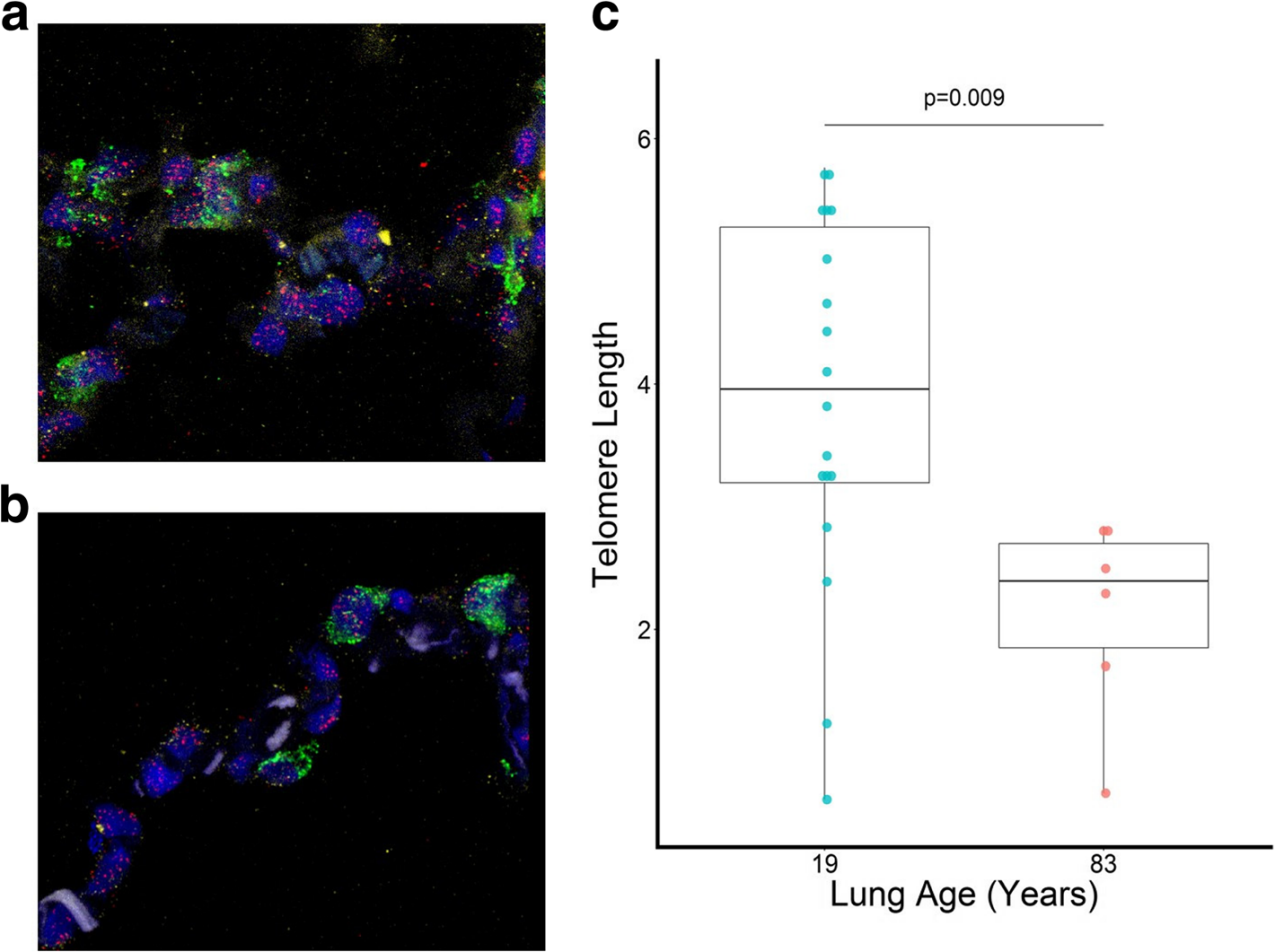
Association of age and telomere length in AT2 cells of normal lung tissue by fluorescent in situ hybridization. Telomere length determination by FISH on normal lung tissue of (**a**) a 19-year old and (**b**) an 83-year old donor showed (**c**) significantly higher telomere length in AT2 cells of the youngest subject (*p* = 0.009). The fluorecent labelling in the **a** and **b** panel stands for green: proSPC (AT2 cells), red: telomere probe, blue: DAPI. Data are presented as boxplots and every dot represents one cell

In order to confirm the association between age and lung tissue telomere length, we performed FISH on two additionally collected donor lungs of opposite age (19 versus 83 years). Results show a clearly higher telomere length in AT2 cells of the youngest donor (*p* = 0.009) (Fig. 2).

### Relative telomere length in lung disease

The range of lung age was dependent on underlying disease (Table 1). The relation between lung tissue RTL and age is shown per disease in Table 2. In BOS and RAS, RTL decreased significantly with every 1-year increase in donor lung age (*p* = 0.022 and *p* = 0.027 respectively). This is in contrast with COPD, where a significant increase in RTL was observed with every 1-year increase in age (*p* = 0.0016).

When affected lung tissue was compared with normal lung tissue, RTL was − 28.0% shorter in cHP tissue (p < 0.0001), − 14.6% shorter in BOS tissue (*p* = 0.0051) and − 10.4% shorter in RAS tissue (*p* = 0.030) in an age, sex and BMI-adjusted model. CF and COPD tissue was not different from normal lung tissue in the same model (Table 4).

**Table 4:** Comparison between RTL in diseased and normal lungs, adjusted for lung age, BMI and sex

Comparison	Estimate	% change	95% CI	*p*-value
CF vs normal	0.0090	2.09	−9.20 - 14.77	0.73
COPD vs normal	0.032	7.67	−2.93 – 19.44	0.16
cHP vs normal	−0.14	−27.97	−34.63 - -20.64	**< 0.0001**
BOS vs normal	−0.069	−14.62	−23.53 - -4.69	**0.0051**
RAS vs normal	−0.048	−10.43	−18.91 - -1.06	**0.030**

Multivariate mixed linear model comparing diseased lungs with normal lungs with lung as random effect, adjusted for lung age, BMI and sex. Estimates are presented as percentage change (95% CI) in average RTL per group compared to normal lungs

*RTL* relative telomere length, *95% CI* 95% confidence interval, *CF* cystic fibrosis, *COPD* chronic obstructive pulmonary disease, *cHP* chronic hypersensitivity pneumonitis, *BOS* bronchiolitis obliterans syndrome, *RAS* restrictive allograft syndrome, vs versus

*p*-value < 0.05 is captured in bold

**Fig. 3:**
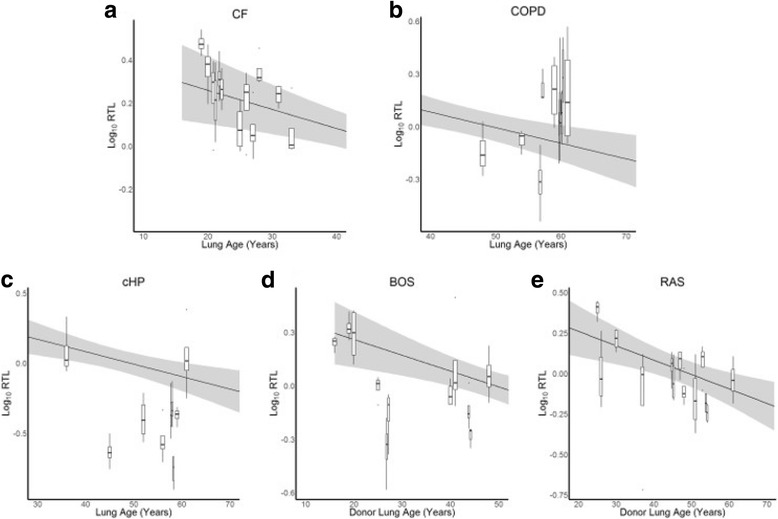
Relation between RTL and age in lung disease. Log_10_ RTL per lung is presented as a boxplot. The grey area in each graph represents the 95% confidence interval of log_10_ RTL in normal lungs. **a**: log_10_ RTL versus lung age in CF lungs. **b**: log_10_ RTL versus lung age in COPD lungs. **c**: log_10_ RTL versus lung age in cHP lungs. **d**: log_10_ RTL versus lung age in BOS lungs. **e**: log_10_ RTL versus lung age in RAS lungs

Figure 3a-e and Table 3 show that in diseased lungs, RTL had considerable variability, both between lungs and within the same lung. A difference in RTL between regions or lobes could not be demonstrated in the diseased lungs, as was observed in the normal lungs (Additional file 1).

### Relative telomere length, structural abnormalities and disease severity

**Fig. 4:**
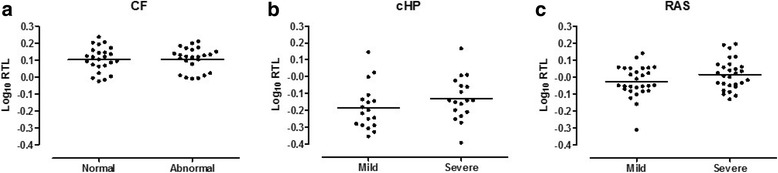
Lack of association between RTL and local disease severity. Every dot represents an individual core log_10_ RTL. Horizontal lines represent means. **a**: RTL in normal versus abnormal CF lung tissue (both *n* = 24), stratified based on HRCT of the lung (*p* = 0.96). **b**: RTL in mildly versus severely affected cHP lung tissue (both *n* = 18), stratified based on surface density of core micro CT (*p* = 0.22). **c**: RTL in mildly versus severely affected RAS lung tissue (both *n* = 28), stratified based on surface density of core micro CT (*p* = 0.084)

Given the low variability in surface density of cores from normal, COPD and BOS lungs, the association between RTL and regional disease severity was not investigated in these lungs. In CF, there was no difference in RTL between tissue cores originating from areas with structural abnormalities on HRCT or normal-appearing tissue (both *n* = 24) (*p* = 0.96) (Fig. 4a). In cHP, RTL did not differ between mildly and severely diseased tissue cores (both *n* = 18) (*p* = 0.22) (Fig. 4b). In RAS, severely diseased tissue cores tended to have longer RTL compared to mildly affected cores (both *n* = 28) (*p* = 0.084) (Fig. 4c). In addition, multivariate analysis demonstrated that for every decrease in surface density of 0.01/μm in RAS tissue, there was an increase in RTL of 1.00% (95% CI: -1.00 to − 0.99, *p* = 0.0027) while accounting for lung age, sex and BMI. The same model could not reveal a significant association between RTL and surface density in CF nor in cHP.

## Discussion

The present study demonstrated an age-dependent RTL decline in normal human lung tissue. In disease-affected lung tissues, the association between lung age and shorter RTL was only present in both CLAD phenotypes. cHP, BOS and RAS tissue had significantly shorter tissue RTL in comparison with normal lungs, when accounting for lung age, BMI and sex. RTL appeared to vary within the studied diseases and within the lung, however we could not demonstrate a link between regional disease severity and shorter tissue RTL.

The association between increasing age and telomere shortening in highly proliferative tissues, such as peripheral blood leucocytes, has been established for quite some time now [21], but knowledge on age-dependent telomere attrition in lung tissue remains scarce. Daniali et al. have shown that there is large variability in telomere length in somatic tissues of the body, which is predominantly established early in life [22, 23]. Highly proliferative tissues, such as blood and skin, have shorter telomeres than minimally proliferative tissues, such as muscle and fat. Nevertheless, it was demonstrated that rates of telomere shortening are similar with increasing age and that telomere length is highly correlated between these tissues [22]. Moreover, a study in primates demonstrated shorter lung telomeres in older animals compared to young ones [24]. Our data confirmed that telomere length does shorten with increasing age in the normal human lung.

Critical telomere shortening due to increasing age or disease may result in cellular senescence [4]. Alveolar type 1 (AT1) and AT2 cells are the most abundant epithelial cells in the lung parenchyma. AT2 cells not only produce surfactant but also serve as progenitor cells for the air-blood barrier forming AT1 cells [4]. Consequently, AT2 cell senescence may explain some of the age-related changes observed in the lungs. In the elderly, an important decline in alveolar epithelial stem cell renewal, compromising the tissue’s regenerative potential, may be observed [25, 26], as well as a pro-inflammatory pulmonary environment maintained by senescent cells via paracrine mediators and promoting further senescence [1, 26]. Finally, alveolar stem cell exhaustion has been discussed as a key factor in age-related pulmonary diseases [27, 28].

In contrast to the findings in normal and CLAD lungs, we could not demonstrate lung age-related RTL decline in CF and cHP lungs. In COPD, the association between tissue RTL and age was even significantly opposite. A few reasons may explain why the association between lung tissue RTL and lung age was not present in each investigated disease. Firstly, the age range was different in each group and was clearly wider in normal, BOS and RAS lungs. Moreover, also disease and host-related factors, other than age, may influence telomere dynamics in the lung. Namely, the activity of telomerase or the expression of essential telomerase genes may significantly determine telomere length. The difference in lung RTL along the apical to basal axis in normal lungs may be explained by the difference in ventilation and perfusion of these regions, with more perfusion in basal regions. Moreover, shorter RTL in basal segments may reflect higher cell turnover, caused by mild atelectasis-related injury, as has been postulated as a mechanism for basal predominance of usual interstitial pneumonia [29]. However, this difference in apical versus basal RTL was not present in diseased lungs.

In CF, little is known on telomere biology to date. Fischer et al. found no difference in telomere length in epithelial cells of CF patients compared to controls [30]. With a full-adjusted model, we could also not demonstrate a shorter RTL in CF compared to normal lungs. Previous studies reported shorter peripheral blood LTL in COPD [11, 31, 32] and telomerase mutations have been demonstrated in 1% of COPD patients, which was associated with emphysema in families with autosomal dominant telomere-mediated disease including pulmonary fibrosis [15]. Studies investigating telomere length of COPD airway epithelial cells are less conclusive. Tsuji et al. found that telomere length in AT2 cells was significantly shorter in patients with emphysema compared to non-smokers [33], whereas Birch and colleagues could not demonstrate shorter telomeres in cultured, primary small airway epithelial cells isolated from COPD patients compared to age-matched controls [34]. The results of our study demonstrate even longer tissue RTL in COPD lungs compared to normal lungs. This finding is surprising, although accelerated aging in COPD may be rather explained by other mechanisms than telomere shortening, such as impairment of anti-aging molecules due to oxidative stress [35]. Furthermore, we are aware that patients with COPD that are eligible for transplantation, represent a subgroup with less comorbidities and possibly less systemic inflammation.

In CLAD, telomere dysfunction is increasingly appreciated as a research target. A small study in lung transplant patients found no association of donor peripheral blood LTL or recipient tissue RTL with survival [36]. More recently, Faust et al. reported that short donor peripheral blood LTL was associated with worse CLAD-free survival after transplantation. In addition, lung allografts later progressing to CLAD demonstrated shorter telomeres in endobronchial biopsies taken within the first 90 days post LTx, suggesting that decreased telomere length within the lung may contribute to CLAD [37]. Our results, showing shorter RTL in CLAD versus normal lungs, support this finding in whole-lung tissue. Within CLAD, RTL did not allow us to differentiate between both phenotypes.

Next to BOS and RAS, also cHP lungs exhibited shorter RTL compared to normal lungs. Idiopathic pulmonary fibrosis (IPF), another interstitial lung disease, is the most frequent manifestation of telomerase-associated disease [14] and shorter peripheral blood LTL has been reported in this disease [12]. In families with multiple pulmonary fibrosis patients, IPF or cHP may occur, suggesting that these disorders have common risk factors. Assuming genetic and clinical analogies between cHP and IPF [16], telomere dysfunction may play a bigger role in the pathophysiology of cHP compared to the other end-stage diseases we investigated.

Despite the reported associations of shorter peripheral blood LTL and increased mortality in cHP [16] and CLAD [37], and the association between shorter telomeres and the extent of fibrosis in both peripheral blood LTL of cHP patients [16] and AT2 cells of IPF patients [38], we could not show an association between lung tissue RTL and local disease severity in cHP and RAS. For cHP, the explanation may be that shorter telomeres encompass a sensitivity for other harmful hits required to manifest disease, as has been suggested for telomere-mediated diseases [23]. Surprisingly, severely diseased RAS tissue cores had longer RTL compared to mildly diseased tissue independent of lung age, sex and BMI. A possible explanation is that the evolution from a normal lung allograft to RAS goes very fast compared with the disease progression of the other studied pathologies, with graft loss occurring 0.6-1.5 years after diagnosis [39]. Even though CF lung disease occurs with an upper lobe predominance [40], we did not find shorter RTL in tissue cores originating from the upper lobes, nor did cores coming from areas with CT alterations demonstrate shorter RTL.

Our results of lung tissue RTL do not fully reflect results from previous studies about peripheral LTL in respiratory diseases. The reason is probably the difference in turnover between these cells. In telomere-mediated diseases, high-turnover tissue with shorter telomeres more rapidly lead to telomere dysfunction, causing cellular senescence whereas short telomeres in low-turnover tissue require other genetic or acquired hits to induce telomere dysfunction [23]. Nevertheless, a previous study on telomere length in COPD and α_1_-antitrypsin deficient patients reported a significant correlation between blood and lung tissue telomere length, notwithstanding that blood LTL was significantly shorter compared to lung tissue telomeres, and a healthy control group was lacking [41].

The use of qPCR to measure RTL may be a limitation of this study, although the main advantage is that it is suitable for high-throughput measurements, requiring a small amount of DNA [42]. A high degree of variation is inherent to this technique, due to differential amplification efficiency or variation in measurement between samples [43]. The use of large pieces of lung tissue, representing multiple cell types contributes to the variability since the occurrence of cellular senescence could be tissue- or cell type-specific and telomere shortening may occur at different rates depending on the cell type. Nevertheless, we would like to emphasize that RTL in this study was measured in triplicate with a T/S ratio coefficient of variation of 7.4%, which can be considered normal. Quantitative FISH methods are probably more accurate and allow cell-specific measurements, but are not a suitable option to determine telomere length on a large scale. Nevertheless, we applied this technique to normal lungs tissue of opposite age to confirm the association between telomere length and lung age.

Another limitation is the lack of peripheral blood of lung donors and transplanted patients in order to determine LTL. This would have enabled us to compare the individual’s LTL with tissue-specific RTL and draw conclusions on a possible correlation between both and their respective value as predictors of disease progression and severity. Unfortunately, this study lacks data of explanted IPF lungs, which is -at least partly- a disease of deficient telomere maintenance [14, 44]. Finally, the lungs included in this study were from patients with end-stage disease that had undergone transplantation, representing a highly specific subgroup of patients. While this precludes drawing any conclusions about early disease stages, any association between telomere dysfunction and disease pathology would likely be more pronounced in these severely diseased lungs.

## Conclusion

In conclusion, this is the first study investigating tissue RTL of normal lungs and end-stage CF, COPD, cHP, BOS and RAS lungs. We demonstrated that RTL inversely correlated with lung age in normal, BOS and RAS lungs, was the opposite in COPD, and not correlated in CF. As well, cHP, BOS and RAS lungs had shorter RTL compared to normal lungs, although telomere length was not associated with regional disease severity.

## Additional file

Additional file 1: Supplementary materials, methods and results. (DOCX 21 kb)

## Abbreviations

AT1: Alveolar type 1 cell
AT2: Alveolar type 2 cell
BMI: Body mass index
BOS: Bronchiolitis obliterans syndrome
CF: Cystic fibrosis
cHP: Chronic hypersensitivity pneumonitis
CLAD: Chronic lung allograft dysfunction
COPD: Chronic obstructive pulmonary disease
CT: Computed tomography
CV: Coefficient of variation
DAPI: 4′,6-diamidino- 2-phenylindolefluorescent
FISH: Fluorescent in situ hybridization
HRCT: High resolution computed tomography
ILD: Interstitial lung disease
IPF: Idiopathic pulmonary fibrosis
IQR: Interquartile range
LTL: Leukocyte telomere length
LTx: Lung transplantation
qPCR: Quantitative polymerase chain reaction
RAS: Restrictive allograft syndrome
RTL: Relative telomere length
SD: Standard deviation
T/S ratio: The ratio of telomere copy number to single-copy gene number
