# Supplementary material for: The aging lung: tissue telomere shortening in health and disease
Source: Respir Res. 2018 May 11;19:95. doi: 10.1186/s12931-018-0794-z (PMC5948770; doi:10.1186/s12931-018-0794-z)
Supplement: Supplementary file 1 — Supplementary materials, methods and results. (DOCX 21 kb) [file 12931_2018_794_MOESM1_ESM.docx]

***Additional file 1***

**Materials and methods**

**Diagnosis of pulmonary diseases**

COPD was diagnosed based on an obstructive spirometry with a FEV1/FVC ratio <0.7 and a history of smoking without clinical and radiological evidence of other respiratory disease.

The diagnosis of CF was mainly based on an elevated concentration of sweat chloride and all cases were confirmed with genetic analysis showing a mutation in each parental allele of the cystic fibrosis transmembrane conductance regulator gene.

HP was diagnosed based on extensive patient history, laboratory results, pulmonary function tests, high resolution computer tomography (HRCT) of the thorax, broncho-alveolar lavage cell counts and surgical lung biopsies.

CLAD was defined as a persistent decline in FEV_1_ of ≥20% compared to the two best post-operative values and after exclusion of a specific cause for this lung function, decline such as acute rejection or infection. So far, two phenotypes of CLAD are being recognized: BOS is the obstructive form of CLAD and is characterized by an obstructive pulmonary function defect, accompanied by scattered obliterative bronchiolitis lesions and air trapping on HRCT. RAS represents a restrictive defect with a decrease in total lung capacity (TLC) of ≥10% compared to baseline and concomitant persistent infiltrates HRCT, or, when TLC was not available, a decrease in FVC of ≥20% compared to the two best post-operative values in combination with persistent infiltrates on HRCT. This classification is discussed more in-depth in (1).

**Telomere length measurement**

In brief, the quantification cycle (C_q_) of the telomeric region and the C_q_ of a single-copy gene (*36B4)* were assessed via a telomeric and single-copy gene specific qPCR respectively. All reactions were run on a 7900HT Fast Real-Time PCR System (Applied Biosystems, Lennik, Belgium) in a 384-well format. A 6-point serial dilution of pooled buffy-coat DNA was included to assess PCR efficiency and three inter-run calibrators were included to account for inter-run variability. C_q_-values of the telomere-specific region were normalized relative to the single-copy gene using qBase software (Biogazelle, Zwijnaarde, Belgium). Relative average telomere lengths were expressed as the ratio of telomere copy number to single-copy gene number (T/S ratio) relative to the average T/S ratio of the entire sample set (n=261). Coefficients of variation (CV) within triplicates of the telomere run, single-copy gene run and T/S ratios were 0.80%, 0.55% and 7.4% respectively.

The forward and reverse primers used for the qPCR of the telomere-specific region were 5’- ACACTAAGGTTTGGGTTTGGGTTTGGGTTTGGGTTAGTGT-3’ and 5’- TGTTAGGTATCCCTATCCCTATCCCTATCCCTATCCCTAACA-3’), respectively. The forward and reverse primers for the single-copy gene were 5’- ACACTAAGGTTTGGGTTTGGGTTTGGGTTTGGGTTAGTGT-3’ and 5’- TGTTAGGTATCCCTATCCCTATCCCTATCCCTATCCCTAACA-3’, respectively. The telomere reaction mixture contained 1x QuantiTect SYBR Green PCR master mix (Qiagen, Inc., Venlo, the Netherlands), 2 mM dithiothreitol (DTT), 300 nM forward primer and 900nM reverse primer. The thermal cycling profile for the telomere reaction was as follows: 1 cycle at 95°C for 10 minutes, followed by 2 cycles at 94°C for 15 seconds and 49°C for 2 minutes and 30 cycles at 94°C for 15 seconds, 62°C for 20 seconds, and 74°C for 1 minute and 20 seconds. The single-copy gene reaction mixture contained 1x QuantiTect SYBR Green PCR master mix, 300 nM forward primer and 500 nM reverse primer. Cycling conditions were: 1 cycle at 95°C for 10 minutes for activation of the DNA polymerase, followed by 40 cycles at 95°C for 15 seconds, and 58°C for 1 minute and 20 seconds.

**Fluorescent in situ hybridization**Frozen tissue cores were immersed in 4% formaldehyde-aceton, then dehydrated with ethanol and thereafter embedded in paraffin. Slides of 7µm were taken and boiled in a tris EDTA buffer after deparaffinisation, to induce antigen retrieval. Telomere labelling was performed using a telomere-Cy3 PNA Probe (Panagene, Daejeon, South-Korea). Alveolar type 2 (AT2) cells were labelled with pro-SPC staining (AB3786,1/500,Merck Millipore, Darmstadt, Germany), DNA of the tissue slides was stained using 4’,6-diamidino- 2-phenylindole (DAPI, 25 μg/mL) and protected with Vectashield antifade mounting medium (Vector laboratories, Burlingame, CA, USA).

A Fluorescence microscope (Leica DM 5500B) at high magnification (63x) was used for image capture, with z-stacking (9 focal planes, 0.5µm intervals). To quantify total telomere fluorescent signal (CY3), a specific ImageJ plugin was used (Telometer). In order to correct for partially captured nuclei, caused by the cutting planes, total telomere signal was divided by the DAPI signal (total DNA). All images were taken within 24 hours after staining to prevent DAPI fluorescence fading.

Multiple images per slice were taken and all pro-SPC positive cells were used. Mean relative telomere signal per cell was calculated as the total Cy3 area divided by the DAPI signal per cell.

**RESULTS**

**Log_10_RTL in apical versus basal and upper versus lower lobe per disease group**

|  | **apical** | **basal** | **p-value** | **upper** | **lower** | **p-value** |
| --- | --- | --- | --- | --- | --- | --- |
| **CF** | 0,12 ± 0,059 | 0,091 ± 0,060 | 0,070 | 0,11 ± 0,056 | 0,096 ± 0,056 | 0,41 |
| **COPD** | 0,046 ± 0,099 | 0,0023 ± 0,080 | 0,12 | 0,048 ± 0,091 | 0,0074 ± 0,081 | 0,051 |
| **cHP** | -0,17 ± 0,11 | -0,15 ± 0,14 | 0,54 | -0,16 ± 0,13 | -0,16 ± 0,13 | 0,90 |
| **BOS** | 0,0072 ± 0,12 | -0,0048 ± 0,083 | 0,59 | 0,016 ± 0,14 | 0,00027 ± 0,080 | 0,57 |
| **RAS** | -0,0087 ± 0,087 | -0,0045 ± 0,067 | 0,76 | -0,012 ± 0,085 | 0,0090 ± 0,063 | 0,32 |

Results of paired t-test per disease group, presented as mean ± sd. CF: cystic fibrosis, COPD: chronic obstructive pulmonary disease, cHP: chronic hypersensitivity pneumonitis, BOS: bronchiolitis obliterans syndrome, RAS restrictive allograft syndrome.

**Reference list**

1. Verleden GM, Raghu G, Meyer KC, Glanville AR, Corris P. A new classification system for chronic lung allograft dysfunction. J Heart Lung Transplant. 2014 Feb;33(2):127–33.
